# A necroptosis-related gene signature to predict prognosis and immune features in hepatocellular carcinoma

**DOI:** 10.1186/s12885-023-11168-8

**Published:** 2023-07-14

**Authors:** Kai Zhang, Jinpeng Li, Enwu Yuan

**Affiliations:** Department of Laboratory Medicine, Third Affiliated Hospital of Zhengzhou University, 7 Kangfu Qian Street, Zhengzhou, 450052 Henan People’s Republic of China

**Keywords:** HCC, Necroptosis, Prognosis, Immunotherapy, LGALS3

## Abstract

**Background and Aim:**

Necroptosis plays an important role in hepatocellular carcinoma (HCC) development, recurrence, and immunotherapy tolerance. We aimed to build a new prognostic necroptosis-related gene signature that could be used for survival and immunotherapy prediction in HCC patients.

**Methods:**

We found that necroptosis was associated with HCC progression and survival outcomes and was involved in the immune infiltration of HCC. Multiple bioinformatics methods including WGCNA, LASSO-Cox regression, stepwise Cox regression, and Random Forest and Boruta model analysis, were used to establish a prognostic profile related to necroptosis. The necroptosis-related gene signature was validated in ICGC and GSE14520 datasets.

**Results:**

This five-gene signature showed excellent predictive performance and was an independent risk factor for patients’ overall survival outcome in the three cohorts. Moreover, this signature was an exact predictor using fewer genes than previous gene signatures. Finally, qRT-PCR and immunohistochemical staining investigations were performed in previously collected fresh frozen tumor tissues from HCC patients and their paracancerous normal tissues, and the results were consistent with the bioinformatics results. We found that LGALS3 not only affected the proliferation and migration ability of HepG2 cells but also affected necroptosis and the expression of inflammatory cytokines.

**Conclusion:**

In summary, we established and validated an individualized prognostic profile related to necroptosis to forecast the therapeutic response to immune therapy, which might offer a potential non-apoptotic therapeutic target for HCC patients.

**Supplementary Information:**

The online version contains supplementary material available at 10.1186/s12885-023-11168-8.

## Introduction

Necroptosis is a novel form of lytic cell death distinct from apoptosis, and the immunostimulatory molecules released during its activation, including inflammatory cytokines and damage-associated molecular patterns (DAMP), can spark an intense inflammatory response and trigger the death of infected or damaged cells in vivo [1]. There is growing evidence that multiple stimuli including TNFα/TNFR signaling, hypoxia, radiation, and chemotherapeutic agents can trigger the necroptosis pathway [2, 3]. Although the intracellular signaling pathways involved in the regulation of apoptosis and necroptosis vary widely; for example, MLKL (mixed lineage kinase domain-like pseudokinase) and RIPKs (receptor-interacting protein kinases) are thought to be key regulators of the necroptosis process, whereas caspases, such as caspase-8, are key mediators of intracellular apoptosis, crosstalk between apoptosis and necroptosis was found during signal transduction [4]. In addition to its ability to fight pathogenic infections and induce strong inflammatory responses, necroptosis is involved in the regulation of cell death and neuroinflammation during the pathology of several neurodegenerative diseases, including Alzheimer’s disease and Parkinson’s disease [5]. Necroptosis has also been found to be involved in tumor progression as a two-sided joker in various types of cancers: on the one hand, key mediators of the necroptosis pathway promote migration and immunosuppression; on the other hand, necroptosis plays a tumor-suppressive role in cancer when apoptosis is impaired [6–8]. The above suggests that further exploration of the mechanisms and functions of the necroptosis pathway is necessary given the critical role of necroptosis in cancer progression and migration as well as in cancer immunosurveillance mechanisms, which will contribute to the development of a new therapeutic approach to eliminate apoptosis-resistant cancer cells for cancer patients.

Previous studies have reported that increased necroptosis is significantly related to nonalcoholic fatty liver disease (NAFLD) in humans [9]. Necroptosis-mediated inflammatory cytokines released in the hepatic microenvironment accelerate liver fibrosis in combination with oxidative stress in a mouse model [10]. The epigenetic hepatic microenvironment is closely related to liver tumorigenesis and hepatocytes containing necroptosis-mediated inflammatory cytokines such as Cxcl13, Ccl6, and Ccl8, which can induce hepatocellular carcinoma (HCC) when surrounded by apoptotic hepatocytes in mice [11]. Furthermore, decreased RIPK3 may enhance the accumulation and polarization of M2 tumor-associated macrophages (TAMs) and participate in the reprogramming of TAM lipid metabolism [12]. We, therefore, speculate that increased hepatic necroptosis plays an important role in the prognosis and tumor immune microenvironment (TME) for HCC, and a deeper insight into it can help clinicians explore new targets for tumor immunotherapy. In this study, we confirmed the speculation of a strong link between necroptosis and poor prognosis and immune cell infiltration in HCC patients, and subsequently established and validated an individualized prognostic profile associated with the necroptosis pathway using multiple bioinformatics methods to forecast the therapeutic response to immune checkpoint blockade (ICB) therapy.

## Materials and methods

### Acquisition of public datasets and characterization of Z-scores associated with necroptosis

Transcriptome expression data from TCGA-LIHC, ICGC (LIRI-JP), and GSE14520 datasets were included in this study, and patients’ general information is listed in Table S1. The TCGA-LIHC and ICGC (LIRI-JP) datasets were obtained based on RNA-seq analysis, and we performed log2 (FPKM + 1) transformation to normalize the RNA-seq data. The GSE14520 dataset was obtained based on Affymetrix Human Genome U133A Array, and we used the ‘LIMMA’ package’s normalizeBetweenArrays function to normalize the data. Samples in the three datasets without complete survival data were not included in this study. Genes participating in the necroptosis pathway were identified from the Molecular Signatures Database (https://www.gsea-msigdb.org/gsea/msigdb) and quantified in each HCC sample in the TCGA using a single sample genomic enrichment analysis (ssGSEA) method based on transcriptome analysis data, with Z-score scaling applied to the ssGSEA scores.

### Functional annotation

To illustrate the functional annotations implicated with the necroptosis-associated genes, Kyoto Encyclopedia of Genes and Genomes (KEGG) pathway enrichment analysis and gene ontology (GO) annotation analysis were performed using the “clusterProfiler” package in R software [13–15]. The significance level (P-value) and false-positive rate (FDR) of each signal pathway were calculated using Fisher exact test and multiple comparison test [16].

### Hub gene selection and gene signature construction

Based on transcriptome expression data and necroptosis Z-scores in TCGA, weighted gene coexpression network analysis (WGCNA) was utilized to build a scale-free coexpression network to determine the gene module most important to necroptosis [17]. Moreover, gene significance (GS) was used to quantify the connections between individual genes and necroptosis Z-scores, while module members reflected the correlations between module characteristic genes and gene expression patterns. Genes identified from the module that was most associated with necroptosis Z-scores were evaluated as candidates using the p value threshold of GS < 0.0001 and the significance level of univariate Cox regression of p < 0.01. The least absolute shrinkage and selection operator (LASSO) Cox regression, stepwise Cox regression, and the Random Forest and Boruta (RFB) model were then used to filter the most reliable prognostic candidates. The multivariate Cox relapse coefficient (β) was utilized to produce a risk score based on the notion of combining the following equation directly with the amount of overlapping gene expression. Risk score=∑iCoefficient (mRNAi)*Expression (mRNAi). We finally separated the patients in the three available datasets into two groups based on the optimum hazard score edge. The predictive control and autonomy of the prognostic signature were assessed using ROC analysis, Kaplan‒Meier survival analysis, and Cox relative risk relapse study.

### Tumor-infiltrating immune cells, genetic alterations, and immune checkpoint gene analysis

Multiple approaches were used to determine the abundance ratios of tumor-infiltrating immune cells (TIICs) in the HCC TME, including CIBERSORT [18], TIMER [19], QUANTISEQ [20], and xCELL [21]. The mutation and CNA data of HCC patients were retrieved from TCGA and the changes in genetic variants across various subgroups were examined using the R package “maftools.“ To determine how the prognostic classifier influences immunotherapies, we looked at the relationship between necroptosis Z-scores, risk scores, and three potentially available targeted immune checkpoint genes, PD1, PD-L1, and CTLA4.

### Drug susceptibility analysis

With 574 advanced clinical trials and 216 FDA-approved drugs used for follow-up research, the association between anticancer drug sensitivity and molecules in the model was directly explored in the CellMiner database [22]. The cut-off criteria for tumor-sensitive drugs were an adjusted P value of < 0.001 and a Pearson correlation coefficient of > 0.4.

### Quantitative real-time polymerase chain reaction (qRT‒PCR) and immunohistochemistry staining assay in clinical samples

Twenty previously collected HCC patients’ fresh frozen tumor tissues and their paracancerous normal tissues were chosen as clinical samples for qRT‒PCR investigation. Table S2 contains the primer sequences. Single-blind and unified criteria methods were used by two experienced pathologists to assess the IHC results. A final score of the sum of the expression score (no positive cells = 0, < 10% = 1, 10–50% = 2, positive staining of > 50% = 3) and the intensity score (negative = 0, weak = 1, moderate = 2, strong = 3) was used to distinguish between low (≤ 4) and high (> 4) expression of LGALS3 in HCC and normal tissues.

### Cell culture and siRNA transfection

HepG2 cells were purchased from the American Type Culture Collection and cultured in recommended DMEM (Sangon Biotech, China) with 10% foetal bovine serum (FBS, Sangon Biotech, China) in 100% humidity at 37 °C with 5% CO2. Small interfering RNA (siRNA) (Sense: 5′-GCC ACU GAU UGU GCC UUA UTT-3′; Antisense:5′-AUA AGG CAC AAU CAG UGG CTT-3′ ) and negative control (Sense: 5′- UUC UCC GAA CGU GUC ACG UTT-3′; Antisense: 5′- ACG UGA CAC GUU CGG AGA ATT-3′) were selected to reduce LGALS3 expression in Hep3B cells using lipofectamine™ 3000 transfection reagent (Invitrogen, Carlsbad, USA) according to the manufacturer’s instructions.

### Cell proliferation and migration assay

According to the manufacturer’s instructions, the vitality of cells was determined using the cell counting kit-8 (CCK-8, Sangon Biotech, Shanghai, China). In the CCK-8 experiment, cells were seeded at a density of 1 × 10^5^ cells per well in 96-well cell culture clusters and cultivated for 1 day, 2 days, and 3 days. After culture, 10 µL of CCK-8 solution was applied to each well, and the absorbance was measured with a microplate reader at 450 nm within 4 h. Cells were seeded in 6-well plates and cultivated to approximately 80% confluence in serum-free media before being scratched with a sterile pipette tip for the wound-healing experiment. After removing cell debris with PBS washing, cells were grown in DMEM with 10% FBS for the following 48 h. After photographing the wound width of the cell monolayers, the area of the wound width was measured. This test required three independent duplicates to assure accuracy, and the wound closure rate was determined from images as [1 - (wound area/original wound area)]. After covering Transwell filters with Matrigel, cultured cells were resuspended in 200 µL of serum-free DMEM at a density of 1 × 10^4^ cells per mL and plated into Transwell inserts, and the wells were filled with 500 L of DMEM supplemented with 10% FBS for the Transwell assay. After being cultured at 37 °C for 48 h, cells adhered to the bottom of the Transwell filters were stained with 0.1% crystal violet in PBS for 15 min and counted under microscopy at 200 x magnification.

### Western blotting

HepG2 cells were harvested in RIPA Lysis Buffer, and were lysed by ultrasound treatment (#DH92-IIN; LAWSON). Supernatants were collected and protein concentrations were determined using Coomassie brilliant blue G250 stain (#C8420, Solarbio). Proteins were separated by SDS-PAGE and transferred to a Nylon membrane. Membranes were blocked in 5% Milk/TBS-T for 2 h at room temperature and incubated overnight at 4 °C with primary antibodies and subsequently with HRP-conjugated secondary antibody [16]. Antibodies against MLKL (#26,539), RIPK1(#3493), RIPK3(#10,188) and β-actin (#93,473) were purchased from CST. Necrostatin-1(Nec-1) (HY-15,760), a necrotic apoptosis inhibitor was purchased from MCE.

### Statistical analysis

Quantitative variables were evaluated using an independent-samples t-test. ROC curve analysis and Kaplan‒Meier survival analysis were utilized to examine the prediction performance of survival outcomes using R software (version 4.0.3). A Cox proportional model was used to explore the relationship between a prognostic classifier and survival outcomes, as well as other clinical factors. The results were considered statistically significant when the P value was less than 0.05.

## Results

### Necroptosis was associated with HCC progression and survival outcomes

**Fig. 1:**
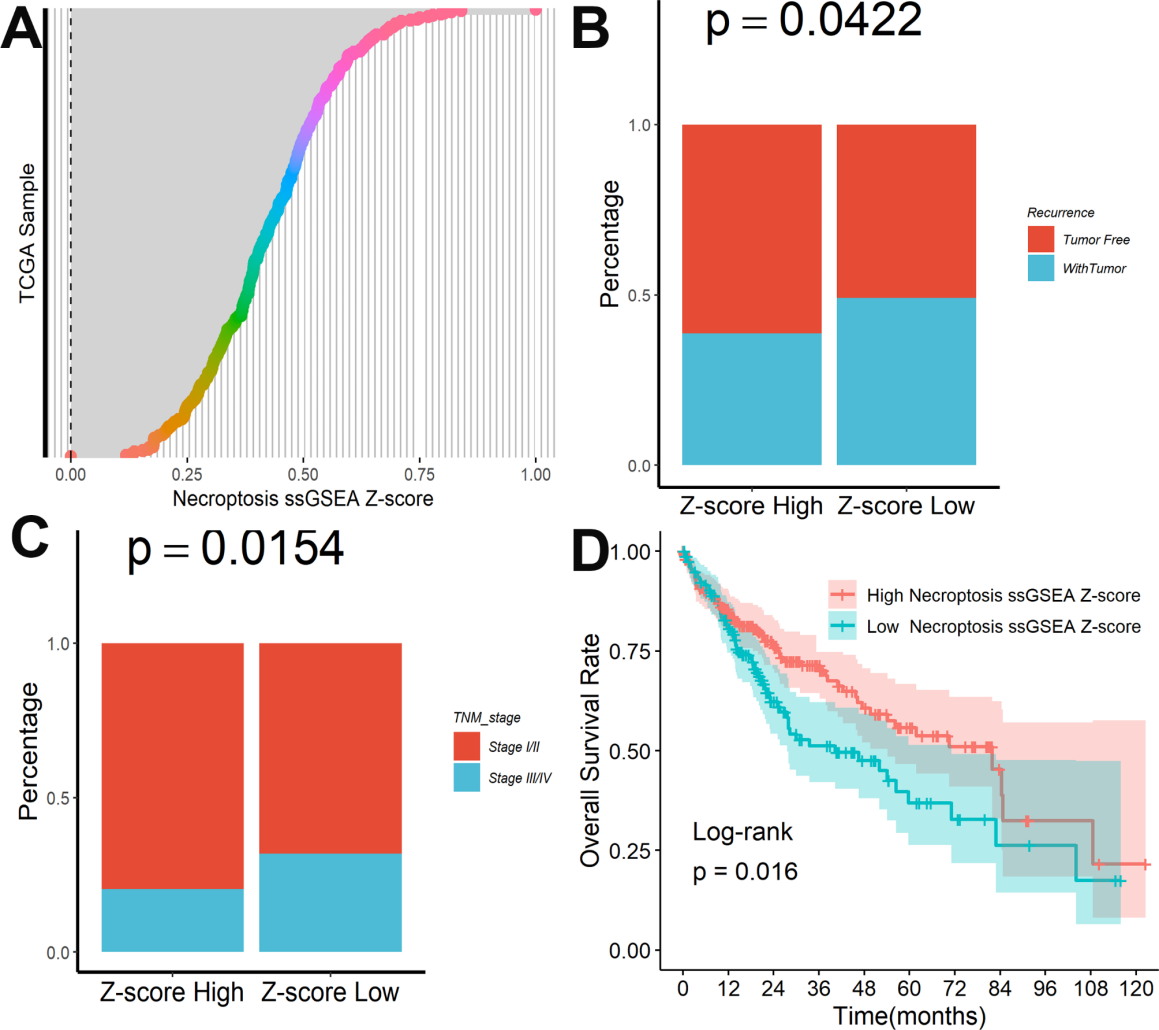
Necroptosis was associated with HCC progression and survival outcomes. **(A)** Overview of the Z-scores associated with necroptosis in TCGA. Differences in the percentage of tumor recurrence **(B)** and advanced clinical stages **(C)** between different Z-score groups. **(D)** Kaplan-Meier survival plot of different Z-scores

A total of 159 necroptosis-associated genes were extracted from prior research and quantified in each sample using ssGSEA analysis in TCGA, as shown in Fig. 1A. HCC patients with a low necroptosis Z-score showed a greater recurrence incidence (Fig. 1B) and more advanced clinical stages during follow-up (Fig. 1C). Furthermore, as demonstrated in Fig. 1D, patients with a greater necroptosis Z-score had considerably better survival outcomes.

### Necroptosis was involved in the immune infiltration of HCC

**Fig. 2:**
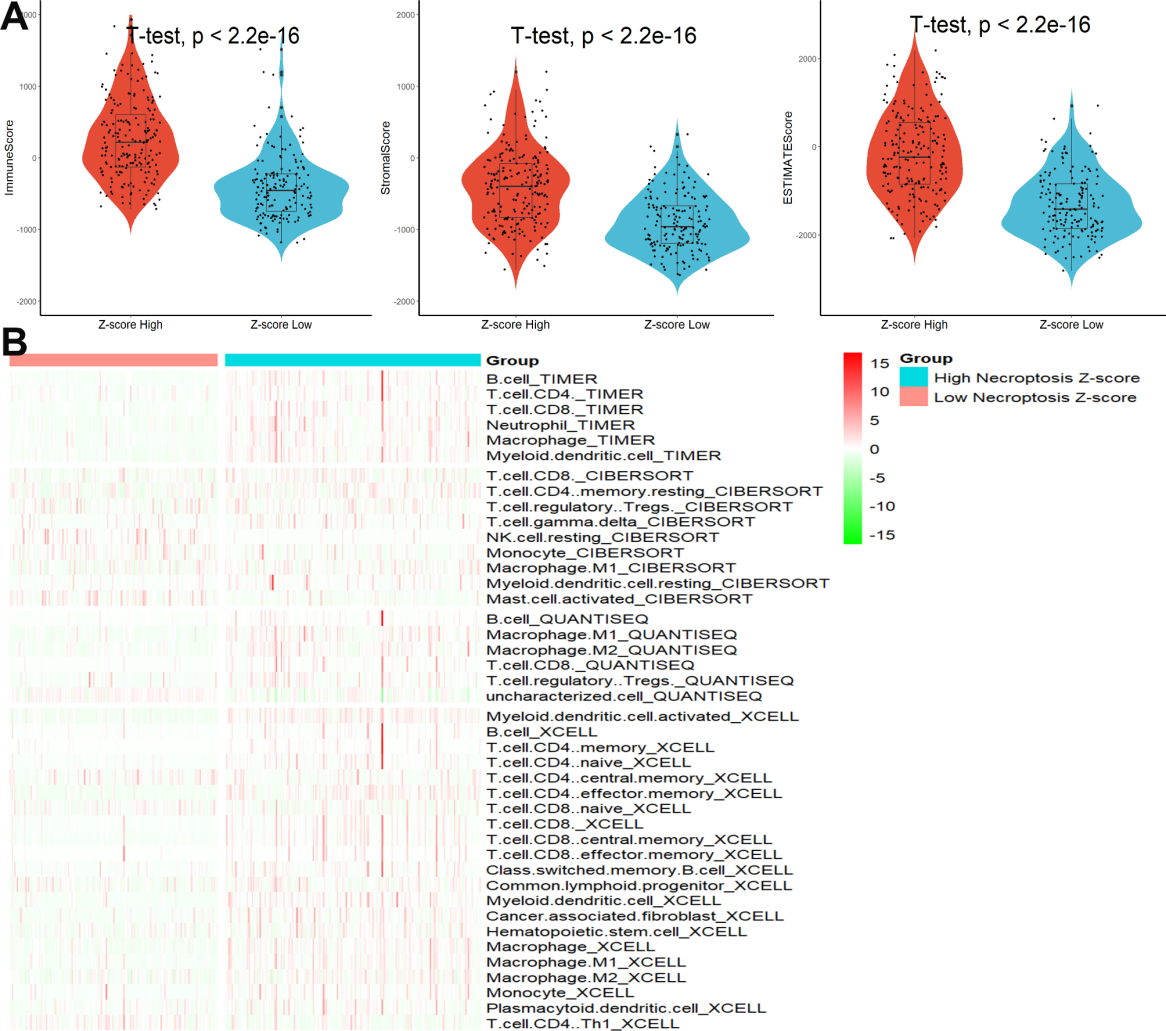
Necroptosis was involved in the immune infiltration of HCC. **(A)** Differences in stromal, immunes, and estimate scores between different Z-score groups. **(B)** Heatmap of levels of immune cell infiltration in different Z-score groups

Stromal and immune fractions were estimated by ESTIMATE to assess the abundance of stromal and immune cells within the tumor [23], and the results showed that HCC samples with high necroptosis Z-score defined as having ‘immune hot’ tumors exhibited higher stromal, immune and ESTIMATE scores (Fig. 2A). Then, a heatmap was plotted to assess the relationship between the necroptosis Z-score and the level of immune cell infiltration, and substantial differences were found. We summarized the composition of the 42 immune cells infiltrating in HCC patients from both high and low necroptosis Z-score groups. The results indicated that high necroptosis Z-score group had a distinct pattern of immune infiltration compared to low necroptosis Z-score group. Furthermore, high necroptosis Z-score group had a significantly higher abundance of immune cells—including memory CD8 T cells, memory B cells, CD4 T cells, TAMs, than low necroptosis Z-score group, but had a significantly lower abundance of immune cells—including resting NK cells, mast cells, than low necroptosis Z-score group (Fig. 2B). Finally, we found that the necroptosis Z-score was significantly related to the expression of the three immune checkpoint genes (Figure S1A), and patients with higher necroptosis Z-scores had remarkably lower expression of PD1, PD-L1, and CTLA4 (Figure S1B).

### Necroptosis-related gene signature construction in TCGA

**Fig. 3:**
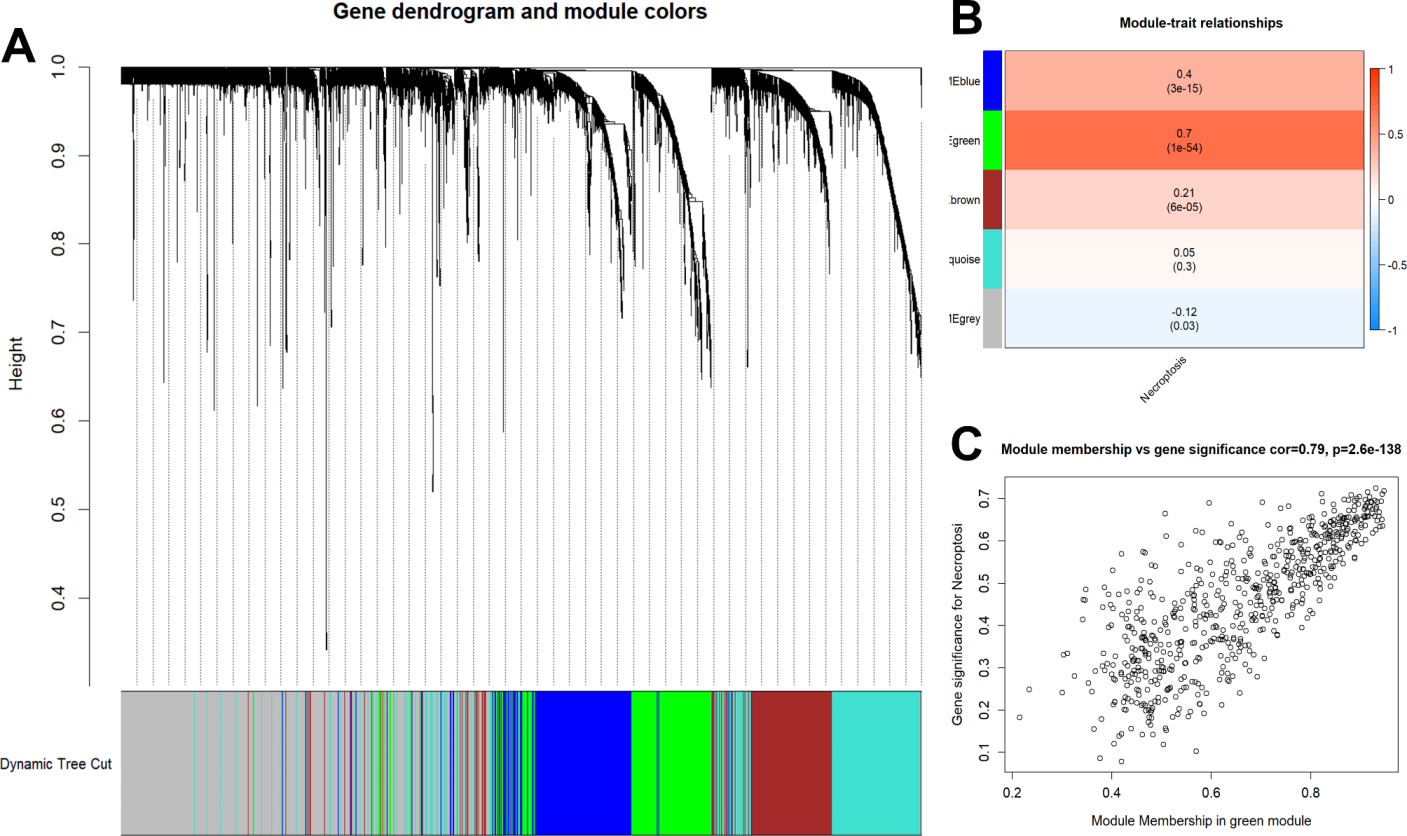
WGCNA for hub genes. **(A)** Four non-gray modules were created in the WGCNA network. **(B-C)** The green module was the most closely associated with necroptosis of the four modules

**Fig. 4:**
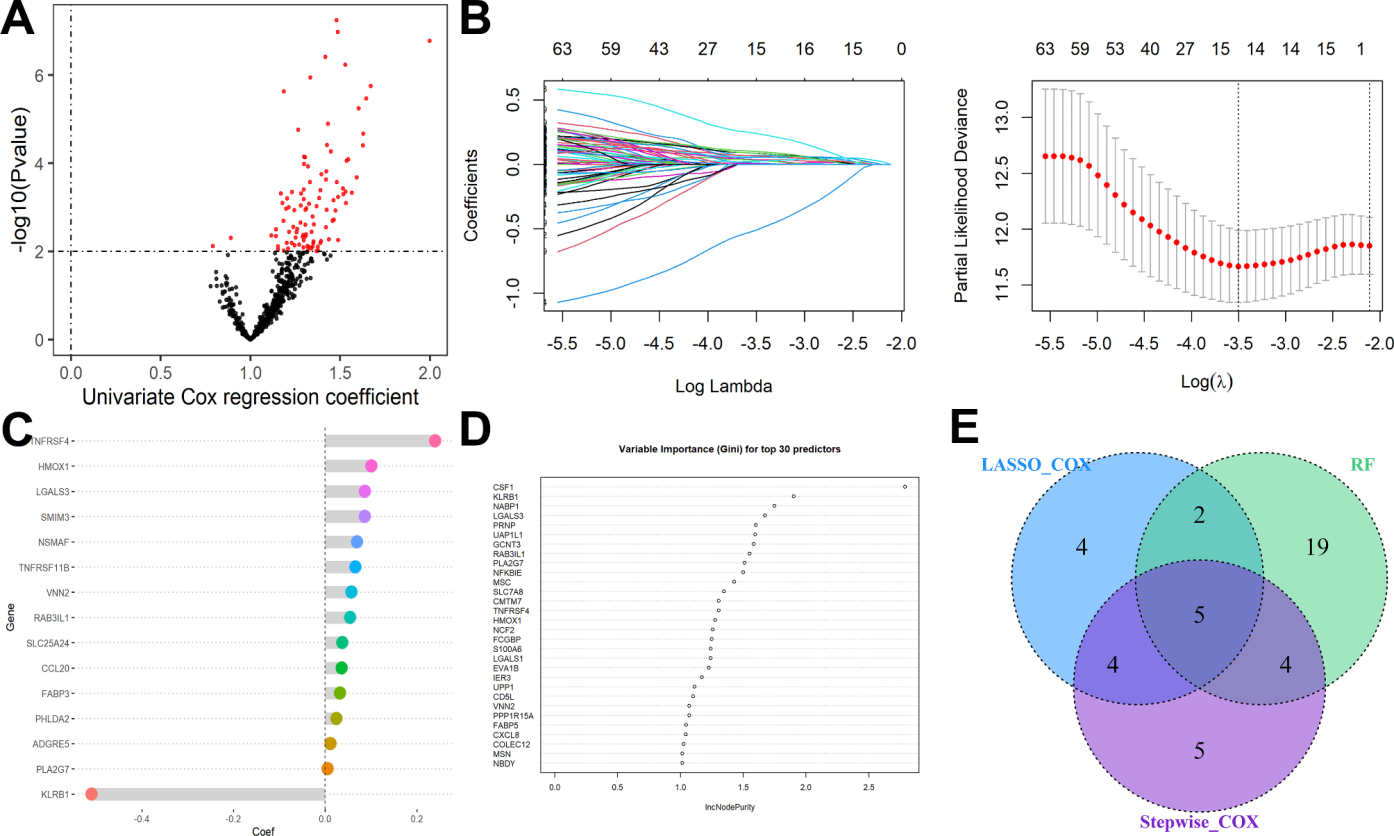
Necroptosis-related gene signature construction in TCGA. **(A)** A total of 81 candidate genes were identified from the green module. **(B)** Genes screened by the LASSO-Cox regression model. **(C)** 15 genes were ruled out. **(D)** The top 30 most important predictors are identified by the RFB model. **(E)** Five overlapping candidate genes were discovered

Following the removal of outliers (Figure S2A), the top 5000 ranked genes with the greatest variation were chosen for WGCNA. A soft threshold power of 9 (Figure S2B) was used to assure the reasonableness of the scale-free network (scale-free R^2^ = 0.9). Four nongray modules were created, as illustrated in Fig. 3A. The green module, which had 639 genes, was the most closely associated with necroptosis of the four modules (Fig. 3B&C). The biological importance of these genes in the green module was investigated using enrichment analysis, and the results revealed that they were mostly enriched in immune activation and response (Figure S3). After that, a univariate Cox regression analysis with P values less than 0.01 was used to look for predictive genes, yielding a total of 81 candidate genes identified (Fig. 4A). The LASSO-Cox regression model was used to further filter these candidate genes (Fig. 4B), and 15 genes were ruled out (Fig. 4C). Similarly, the RFB model was used to filter these candidate genes, and the top 30 most important predictors are shown in Fig. 4D. Eighteen candidate genes were filtered by stepwise Cox proportional analysis. Five overlapping candidate genes were discovered (Fig. 4E) and then incorporated into a multivariate Cox regression model to create a predictive five-gene signature. Risk score = HMOX1*0.3318815 + VNN2*0.1493589 + TNFRSF4*0.4052564 - KLRB1*0.6610017 + LGALS3*0.2188195.

### The necroptosis-related gene signature was an independent risk factor for patients’ overall survival outcome in TCGA

**Fig. 5:**
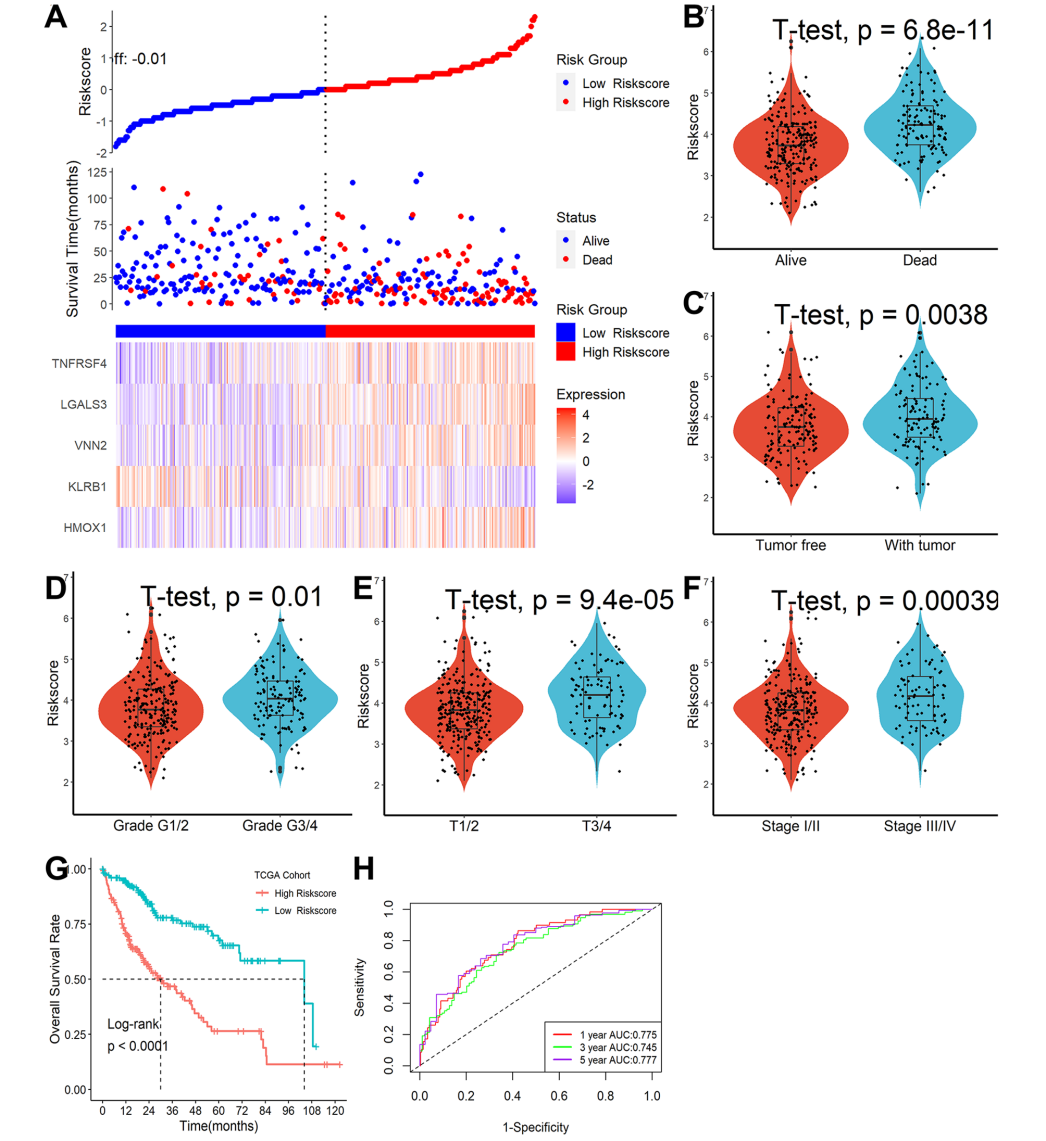
Necroptosis-related gene signature was an independent risk factor for patients’ overall survival outcome in TCGA. **(A)** Distribution of risk scores, OS status, and gene expression profiles. HCC patients with high-risk scores had higher mortality **(B)**, recurrence rate **(C)**, and more advanced clinical stages **(D-F)**. **(G)** Patients with lower risk scores had significantly improved survival results. **(H)** ROC analysis revealed that this necroptosis-related gene signature showed excellent predictive performance

Using an adequate risk score threshold, patients were split into high- and low-risk categories (Fig. 5A). During follow-up, we discovered that HCC patients with high risk scores had higher mortality (Fig. 5B), and recurrence rates (Fig. 5C) and more advanced clinical stages (Fig. 5D-F). Furthermore, patients with lower risk scores had significantly improved survival results, as shown in Fig. 5G. Additionally, ROC analysis revealed that this necroptosis-related gene signature showed excellent predictive performance, with AUCs of 0.775, 0.745, and 0.777 at one, three, and five years, respectively (Fig. 5H). Finally, after adjusting for other clinical features, multivariate Cox regression analysis revealed that this signature could serve as an independent prognostic factor for HCC patients (HR = 2.278, 95% CI 1.590–3.263, P < 0.001).

### The necroptosis-related gene signature was also an independent risk factor for patients’ overall survival outcome in the ICGC and GSE14520 datasets

**Fig. 6:**
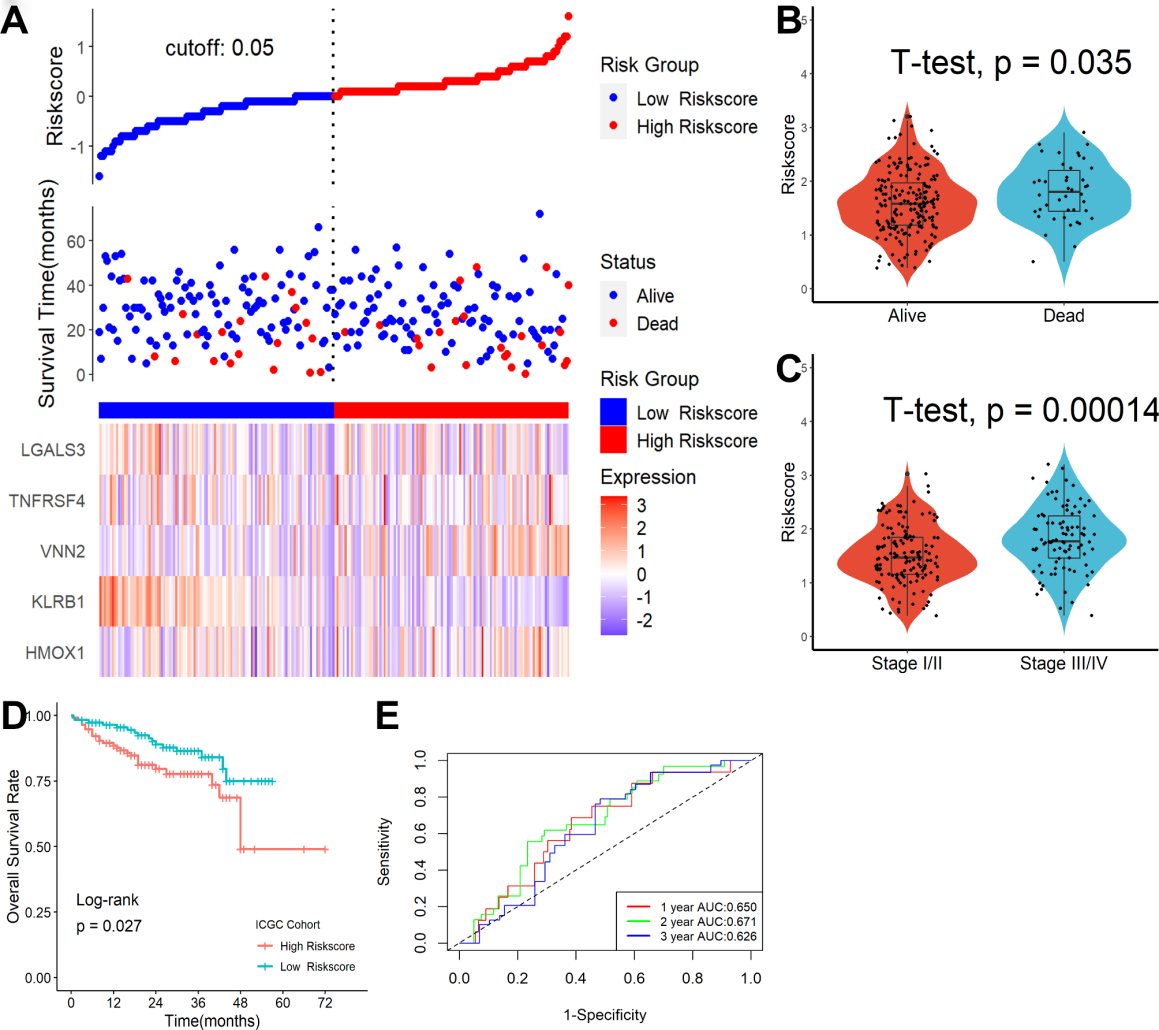
Necroptosis-related gene signature was an independent risk factor for patients’ overall survival outcome in the ICGC dataset. **(A)** Distribution of risk scores, OS status, and gene expression profiles. HCC patients with high-risk scores had higher mortality **(B)** and more advanced clinical stages **(C)**. **(D)** Patients with lower risk scores had significantly improved survival results. **(E)** ROC analysis revealed that this necroptosis-related gene signature showed excellent predictive performance

**Fig. 7:**
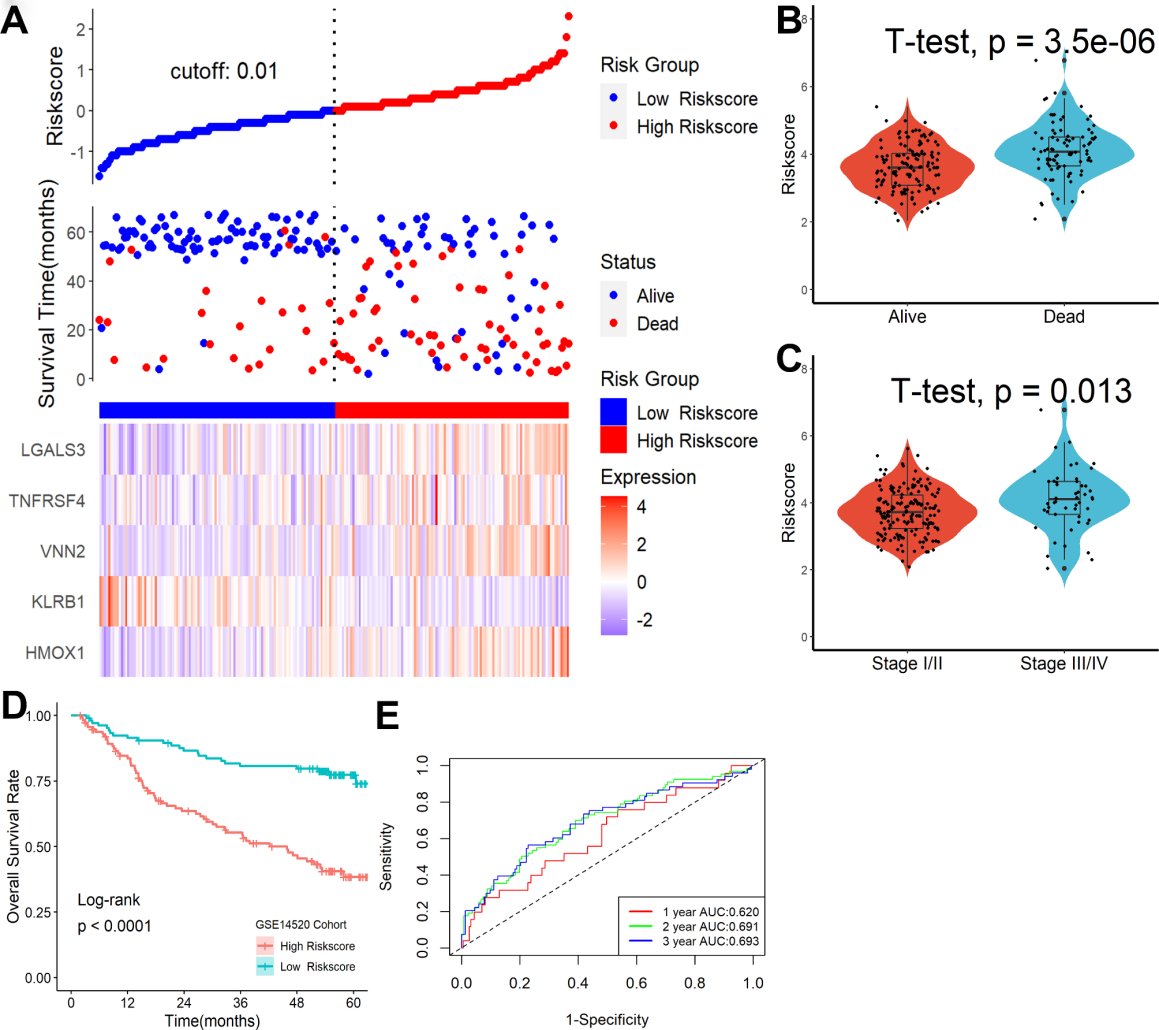
Necroptosis-related gene signature was an independent risk factor for patients’ overall survival outcome in the GSE14520 dataset. **(A)** Distribution of risk scores, OS status, and gene expression profiles. HCC patients with high-risk scores had higher mortality **(B)** and more advanced clinical stages **(C)**. **(D)** Patients with lower risk scores had significantly improved survival results. **(E)** ROC analysis revealed that this necroptosis-related gene signature showed excellent predictive performance

To validate the signature, the ICGC and GSE14520 datasets were utilized as validation cohorts. In the ICGC cohort, patients were split into high- and low-risk categories by using an adequate risk score threshold (Fig. 6A). During follow-up, we discovered that HCC patients with high-risk scores had higher mortality (Fig. 6B) and more advanced clinical stages (Fig. 6C). As shown in Fig. 6D, patients with lower risk scores had significantly improved survival results. Moreover, ROC analysis revealed that this necroptosis-related gene signature showed good predictive performance (Fig. 6E). Similarly, patients in the GSE14520 dataset were split in to high- and low-risk categories by using an adequate risk score threshold (Fig. 7A). HCC patients with high-risk scores had higher mortality (Fig. 7B) and more advanced clinical stages (Fig. 7C) during follow-up. As seen in Fig. 7D, patients with higher risk scores had significantly poorer survival results. Additionally, ROC analysis showed that this necroptosis-associated genetic signature was an exact predictor of the overall survival rate (Fig. 7E). Most importantly, multivariate Cox regression analysis revealed that this signature could also serve as an independent prognostic factor for HCC patients in ICGC (HR = 2.278, 95% CI 1.590–3.263, P < 0.001) and GSE14520 (HR = 2.278, 95% CI 1.590–3.263, P < 0.001) after adjusting for other clinical features.

### Genetic alterations and immune checkpoint gene analysis

As shown in Figure S4, the mutation rates of the top 10 most substantially altered genes were significantly different between the high- and low-risk score subgroups, according to the results of genetic alteration analysis. Furthermore, we found that risk scores were significantly related to the expression of the three immune checkpoint genes (Figure S5A) and patients with higher risk scores had remarkably higher expression of PD1, PD-L1, and CTLA4 (Figure S5B).

### The necroptosis-associated gene signature was an exact predictor using fewer genes

Comparison of the predictive power of multiple genetic signatures can help to further explore the prognostic value of these genetic signatures. Therefore, we compared the predictive ability of the following five gene signatures: (1) the necroptosis-associated-gene signature constructed in this study; (2) the six-gene signature constructed by Liu et al. [24]; (3) the ten-gene signature constructed by Liang et al. [25]; (4) the twelve-gene signature constructed by Ouyang et al. [26] and (5) the fourteen-gene signature constructed by Zhang et al. [27]. As shown in Figure S6, our five-gene signature has the best survival prediction ability with fewer genes.

### Building a Nomogram Model

The coefficient prediction efficacy of this necroptosis-associated-gene signature was investigated using a nomogram model in the TCGA dataset, and nomogram construction and validation were performed in accordance with the nomogram guidelines [28, 29]. The findings revealed that the nomogram might help us provide a quantitative approach for appropriately predicting the 1-, 3-, and 5-year survival rates (Figure S7A). The calibration curves revealed that the expected and actual likelihood of 1-, 3-, and 5-year survival rates were in good agreement (Figure S7B).

### Expression levels of genes in the necroptosis-associated gene signature

**Fig. 8:**
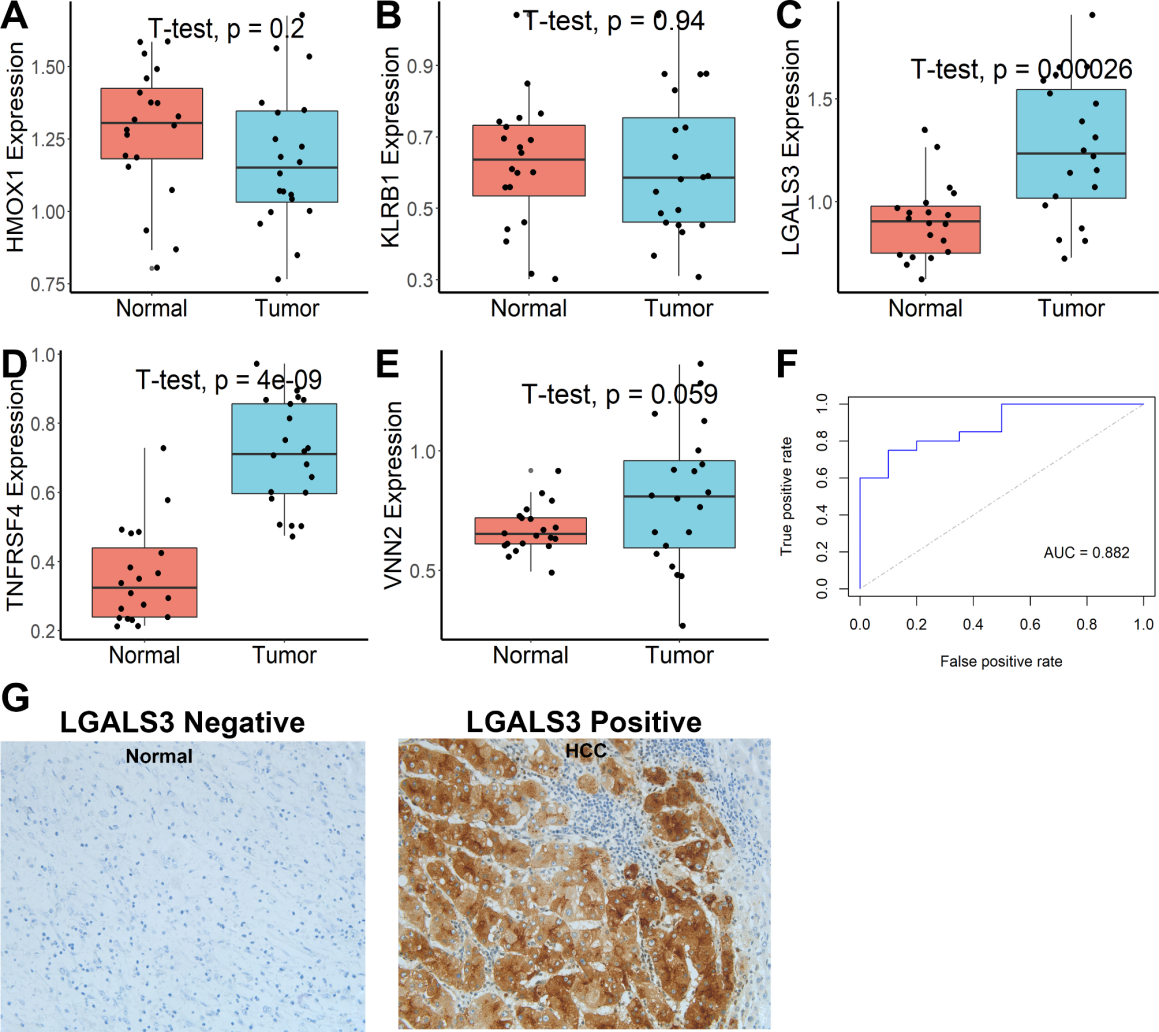
Expression levels of the five genes analyzed by qRT-PCR and IHC assay. **(A-E)** Expression levels of the five genes between tumor tissues and their paracancerous normal tissues. **(F)** Scores in clinical samples could distinguish well between tumor and normal tissue. **(G)** LGALS3 was remarkably elevated in tumor tissues and increased with the progression of HCC.

Gene Expression Profiling Interactive Analysis (GEPIA) [30] was used first to investigate the expression levels of the five genes, and the results showed that only TNFRSF4 and LGALS3 varied substantially between normal and tumor samples (Figure S8A). Then, using the Human Protein Atlas database (HPA) [31], the protein expression of the five genes was investigated, and differences in protein levels were detected between HMOX1 and LGALS3 (Figure S8B). In addition, the expression levels of the five genes in HCC cell lines (Supplementary Figure S8C) were investigated using the Cancer Cell Line Encyclopedia (CCLE) database [32]. Finally, in clinical samples, qRT‒PCR and IHC assays were performed to corroborate the expression of genes in our model. As shown in Fig. 8A-E, TNFRSF4 and LGALS3 varied substantially between tumor tissues and their paracancerous normal tissues. Moreover, the scores obtained according to the formula in clinical samples could distinguish well between tumor and normal tissue (AUC = 0.882, Fig. 8F). Considering that only TNFRSF4 and LGALS3 differed in clinical samples and that no protein expression of TNFRSF4 was detected in the HPA database, we performed immunohistochemical staining analysis only for LGALS3. As shown in Fig. 8G, LGALS3 was remarkably elevated in tumor tissues.

### Effect of LGALS3 knockdown on the proliferation and migratory capacity of HepG2 cells and the expression of key regulatory genes of necroptosis and inflammatory cytokines

**Fig. 9:**
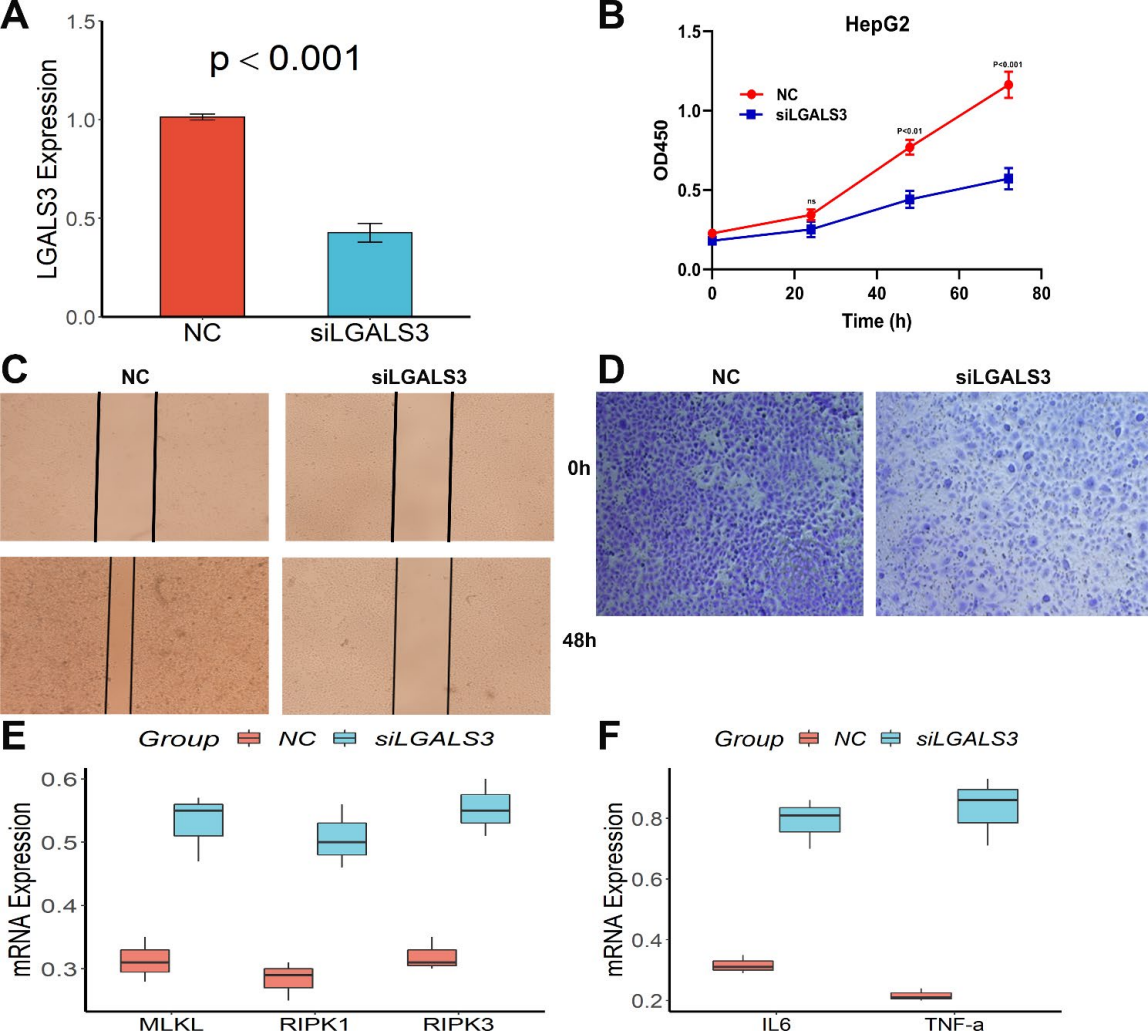
Knockdown of LGALS3 inhibited HepG2 cells proliferation and migration and increased the expression of key regulatory genes of Necroptosis and inflammatory cytokines. **(A)** Decreased LGALS3 in HepG2 cells. **(B)** CCK-8 assay. **(C)** Wound-healing assay. **(D)** Transwell assay. **(E)** and **(F)** Increased MLKL, RIPK1, RIPK3, IL6, and TNF-α levels after knockdown of LGALS3.

In HepG2 cells, the siRNA significantly reduced LGALS3 expression, as shown in Fig. 9A. CCK-8 analysis revealed that downregulated LGALS3 had a lower proliferation rate (Fig. 9B), and wound-healing experiments revealed that LGALS3 knockdown significantly slowed wound closure (Fig. 9C). LGALS3 knockdown significantly decreased cell invasion in transwell chambers using the Matrigel test (Fig. 9D). Furthermore, knocking down LGALS3 greatly elevated the expression of three important necroptosis regulating genes (MLKL, RIPK1, and RIPK3, Fig. 9E), as well as the levels of inflammatory cytokines such as IL10 and TNF-α (Fig. 9F). Sametime, in order to further determine the protein expression of necroptosis regulating genes, western blot was performed LGALS3 knockdown and co-culture with necrotic apoptosis inhibitor (Nec-1). This was consistent with our expectation that MLKL, RIPK1, and RIPK3 were high expression in HepG2 cell line after LGALS3 knockdown but were significantly decreased after treatment with Nec-1(Figure S10).

### Drug susceptibility analysis

Based on the results of drug sensitivity studies, 21 drugs were found to be tumor-sensitive, and the top 16 most important tumor-sensitive drugs are depicted in Supplementary Figure S9.

## Discussion

Necroptosis is a cellular response to inflammation or infection that is distinct from apoptosis and regulated by the TNF-α receptor system [4]. Cells that die from apoptosis or necrosis have different effects on inflammation within the surrounding microenvironment. The plasma membrane of cells that die due to apoptosis remains intact and has a low inflammatory response around it; however, the plasma membrane of cells dying due to necroptosis ruptures, and the released intracellular contents directly activate and modulate the inflammatory response. There is growing evidence that necroptosis is involved in many states of pathology, including stroke [33], neurodegenerative diseases [5], myocardial infarction [34], liver or kidney injury [35, 36], pancreatitis [37], autoimmune diseases [38] and even cancers [39]. Considering that the expression of the major mediators of necroptosis can be regulated by gene deletion or modification in mouse models with no effect on mouse development [40], necroptosis is expected to be the next target for immunotherapy.

Previous studies have shown that intratumor necroptosis can be induced by hypoxia, malnutrition, chemotherapeutic or biological agents (such as cisplatin and bortezomib), or pro-necrotic stimulating factors secreted by tumor-infiltrating immune cells [41–43]. DNA methylation progressively suppresses RIPK3 protein expression during tumor progression to avoid necroptosis, while DNA hypomethylating agents can rescue the necrotic capacity of RIPK3 and promote the efficacy of antitumor immunity [44]. In addition, the release of DAMPs and cytokines in the TME by cancer cells in the necrotic state can facilitate the interaction between dying cancer cells and immune cells, thereby mediating the immune response associated with cancer [45]. Although several studies have confirmed the impact of necroptosis on the prognosis of cancer patients, the conclusions are inconsistent and may be related to tumor heterogeneity, cancer type, and different origin organs of the tumor [46]. Interestingly, methylation-dependent deletion of RIPK3 expression significantly inhibited necroptosis in a variety of common human hepatoma cell lines [44]. Sabira reported that increased necroptosis, which is closely associated with liver ageing, could cause chronic inflammation and promote liver fibrosis [47]. Chen found that heparinase could facilitate HCC metastasis by triggering necroptosis of microvascular endothelial cells [48].In contrast, Silvano reported that increased necroptosis by overexpressing SerpinB3 was beneficial in improving the survival of HCC patients [49]. Given the controversial prognostic role of necroptosis in oncology, there is an urgent need for a specific biomarker or spectrum technology that can consistently and specifically identify necroptosis in complex human tumor tissues.

In this study, after Z-scores of necroptosis were identified by the ssGSEA algorithm, they were shown to be strongly related to patient survival outcomes and involved in the immune infiltration of HCC. Subsequently, we established and validated an individualized prognostic profile associated with the necroptosis pathway using multiple bioinformatics methods to forecast therapeutic responses to ICB therapy. Moreover, when compared with previous signatures, our necroptosis-related gene signature had the best survival prediction ability with fewer genes. Among the five genes, HMOX1 was an unfavorable predictor of HCC prognosis [50], and its expression was also correlated with the efficacy of immunotherapy, such as sorafenib [51, 52]. In addition, HMOX1 was also associated with post-transplant HCC recurrence [53]. VNN2 is a gene related to hydrolase activity that has been identified as a prognostic indicator for HCC patients [54]. The TNFRSF4-NF-κB pathway is considered to be a promising therapeutic target for immune-related gene dysregulation in HCC [55]. High expression of KLRB1 is closely associated with early HCC relapse, and it was also involved in CD8 + T-cell associated immune escape mechanisms [56]. LGALS3 promotes tumor metastasis by activating the β-catenin signaling pathway in HCC, and it is also a reliable biomarker for predicting sorafenib resistance [57]. In this study, we found that LGALS3 not only had an effect on the proliferation and migration ability of HepG2 cells but also affected necroptosis and the expression of inflammatory cytokines.

Our study has certain limitations. First, individual variability in HCC patients may alter the expression of this necroptosis-related gene signature, and therefore future prospective multicenter randomized controlled trials are needed to assess the robustness and clinical usability of the five gene signatures. Furthermore, the biological mechanism between this five-gene signature and the necroptosis pathway in HCC needs to be clarified by biological experimental research in the future.

## Conclusions

Overall, we found that necroptosis was associated with HCC progression and survival outcomes and was involved in the immune infiltration of HCC. Subsequently, we established and validated an individualized prognostic profile related to necroptosis using multiple bioinformatics methods to forecast the therapeutic response to immune therapy, and found that LGALS3 affected necroptosis, which might offer a potential nonapoptotic therapeutic target for HCC patients.

## Electronic supplementary material

Below is the link to the electronic supplementary material.

Supplementary Material 1

Supplementary Material 2

## Data Availability

The datasets used and/or analyzed during the current study are available from the corresponding author on reasonable request.
