# Supplementary figures and images for: A necroptosis-related gene signature to predict prognosis and immune features in hepatocellular carcinoma
Source: BMC Cancer. 2023 Jul 14;23:660. doi: 10.1186/s12885-023-11168-8 (PMC10347745; doi:10.1186/s12885-023-11168-8)

β-actin


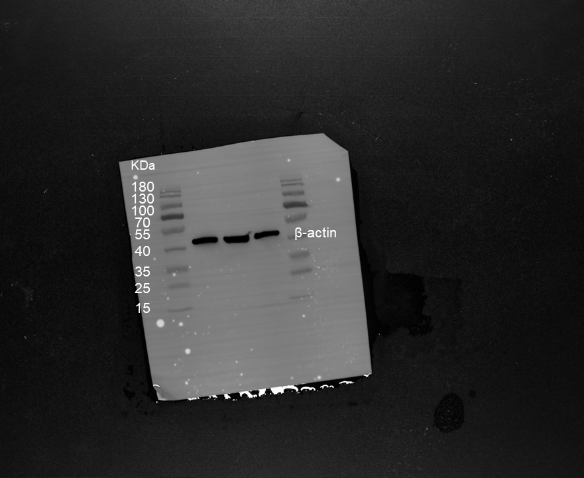




MLKL


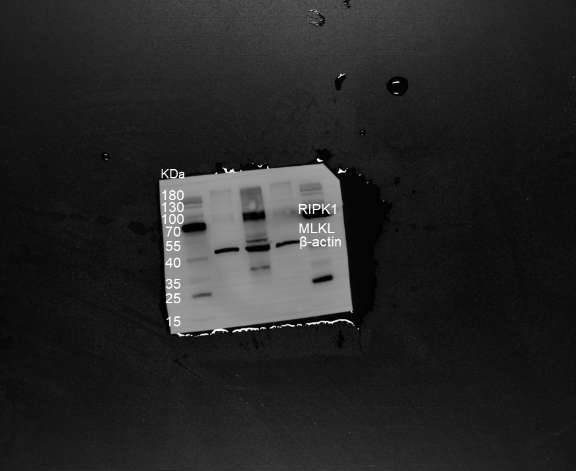




RIPK1


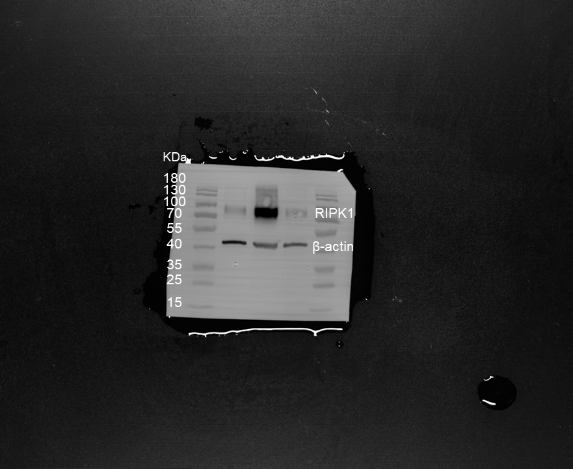




RIPK3


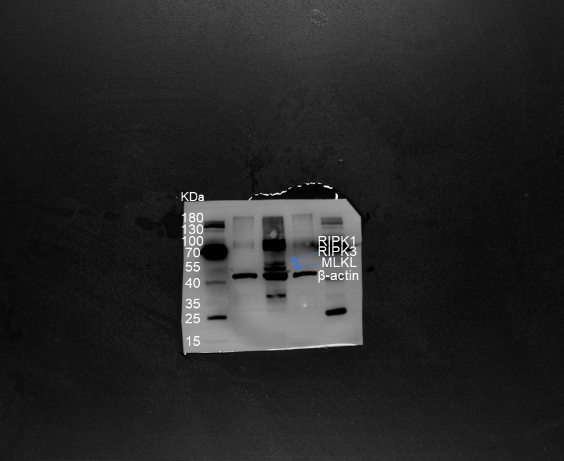

Supplement: Supplementary file 1 — Supplementary Material 1 [file 12885_2023_11168_MOESM1_ESM.docx]
