# Supplementary material for: A necroptosis-related gene signature to predict prognosis and immune features in hepatocellular carcinoma
Source: BMC Cancer. 2023 Jul 14;23:660. doi: 10.1186/s12885-023-11168-8 (PMC10347745; doi:10.1186/s12885-023-11168-8)
Supplement: Supplementary file 2 — Supplementary Material 2 [file 12885_2023_11168_MOESM2_ESM.docx]

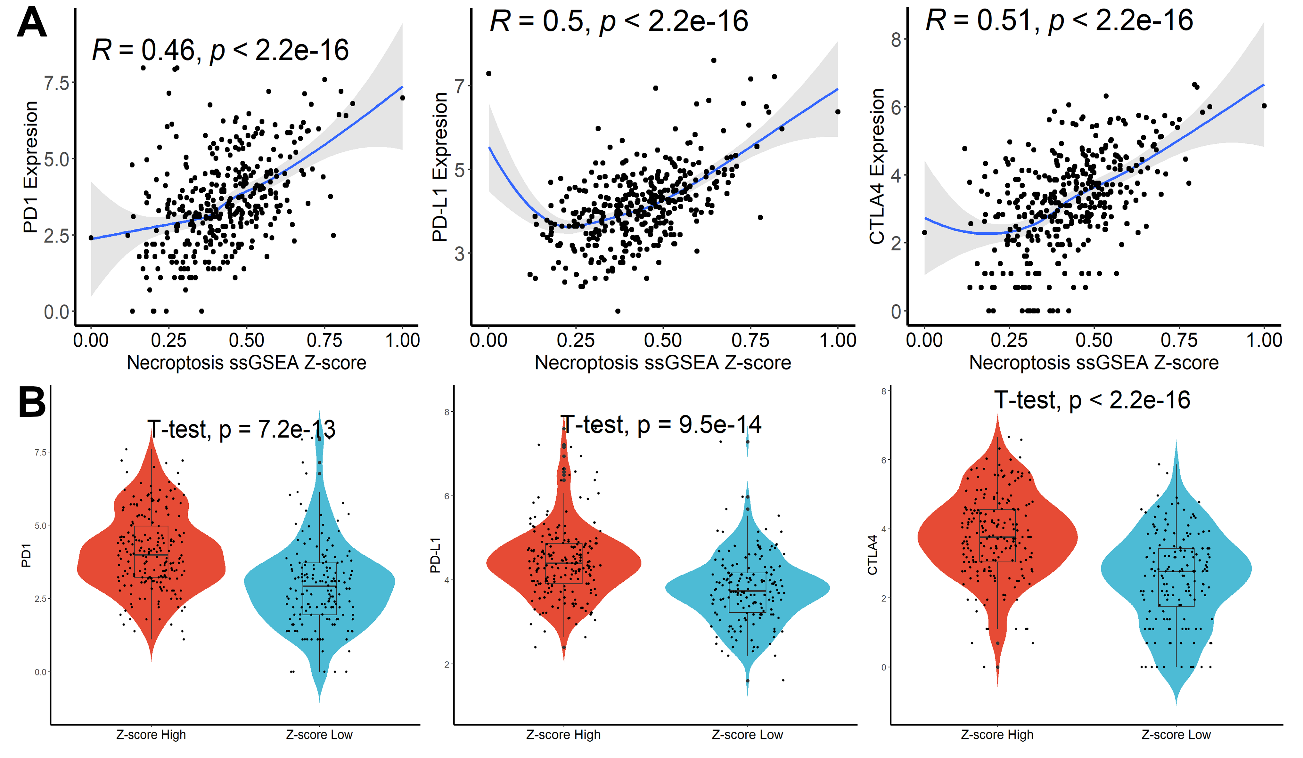


Figure S1 Necroptosis ssGSEA Z-score correlation and differential analysis with PD1, PD-L1, and CATL4 expression.


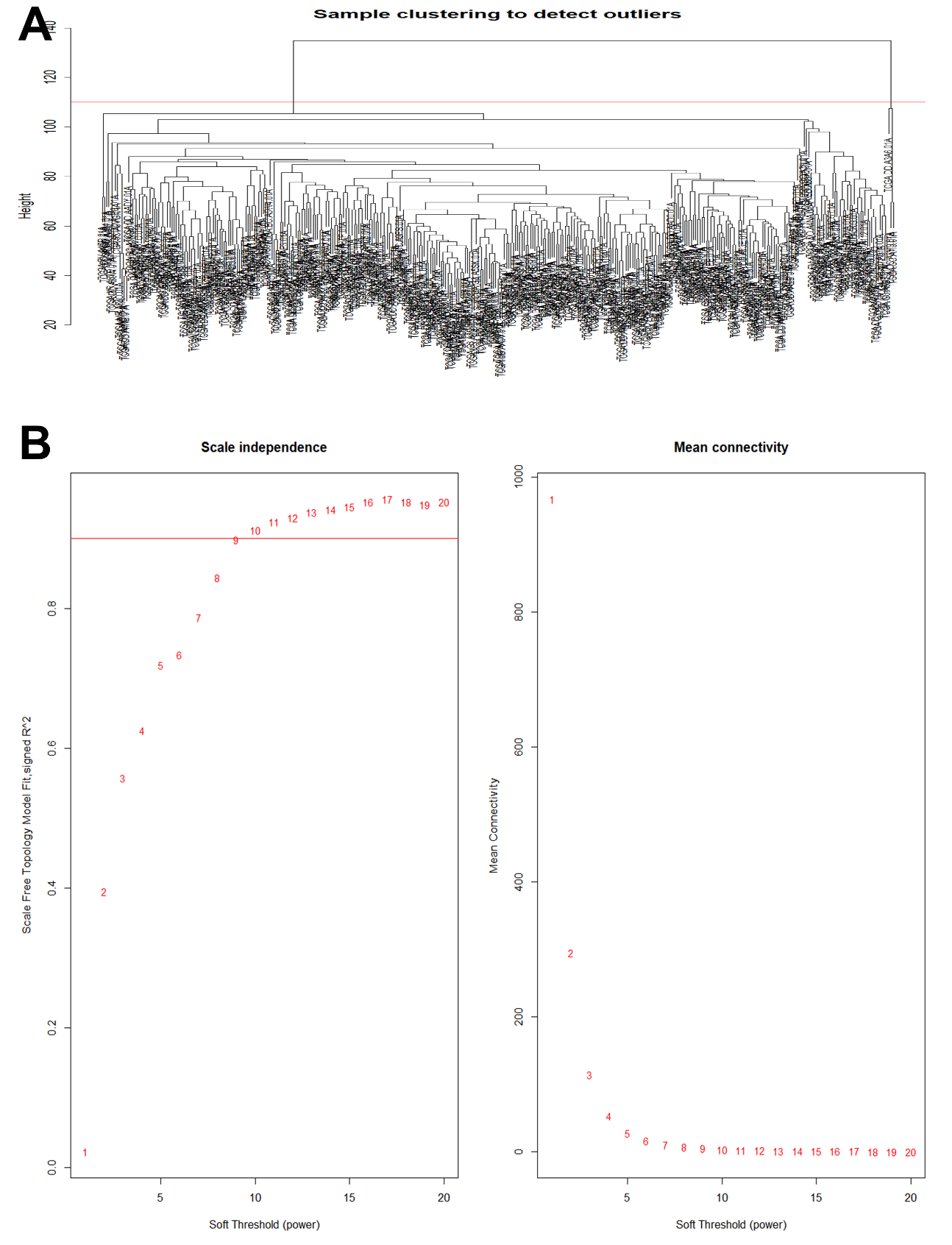


Figure S2 Identify the weighted value β that meets the law of scale-free networks.


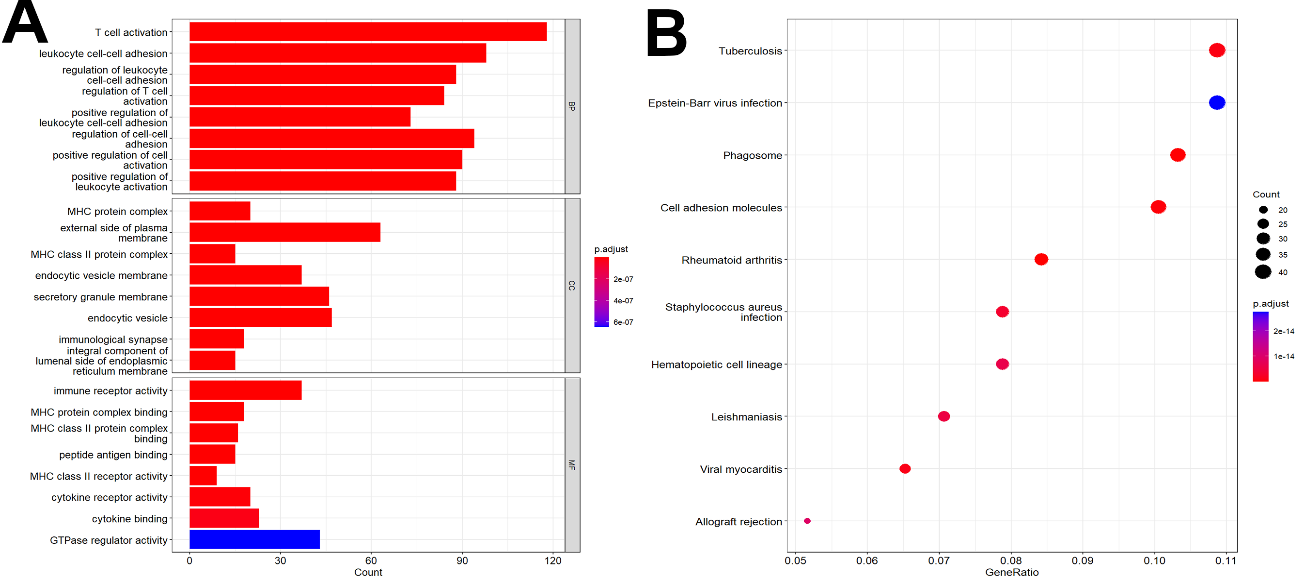


Figure S3 Identification of GO and KEGG enrichment of necroptosis-associated genes.


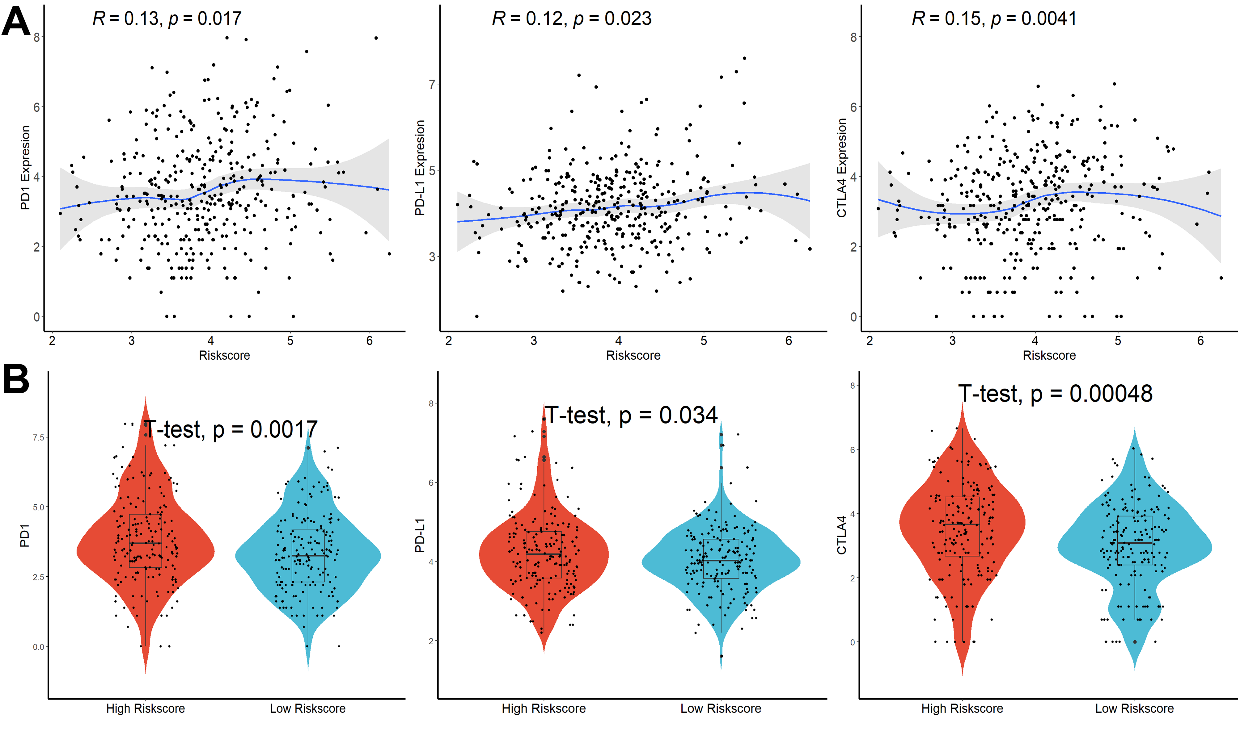


Figure S4 Five-gene prognostic model correlation and differential analysis with PD1, PD-L1, and CATL4 expression.


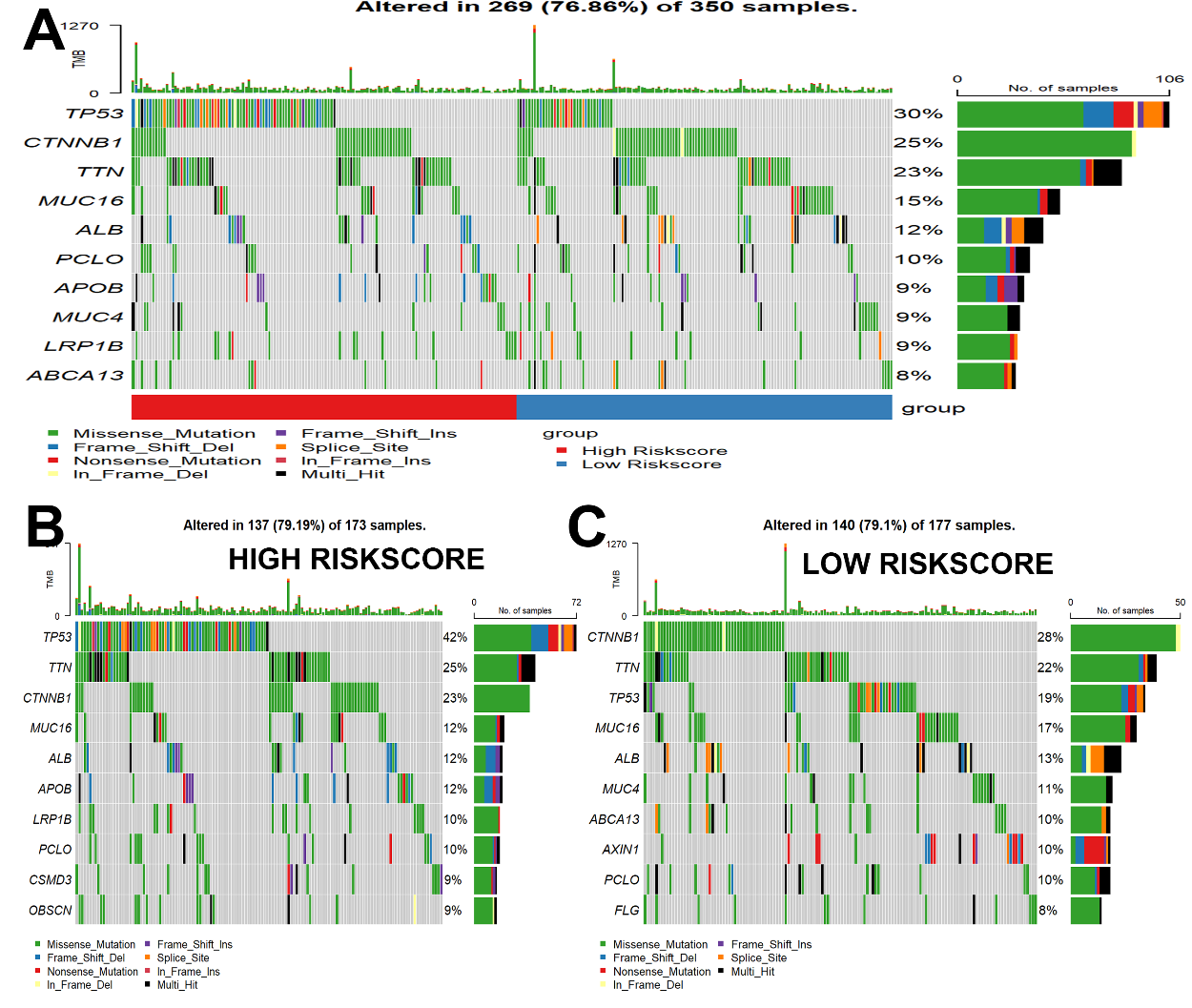


Figure S5 Tumor maps of top ten mutated genes in the five-gene prognostic model.


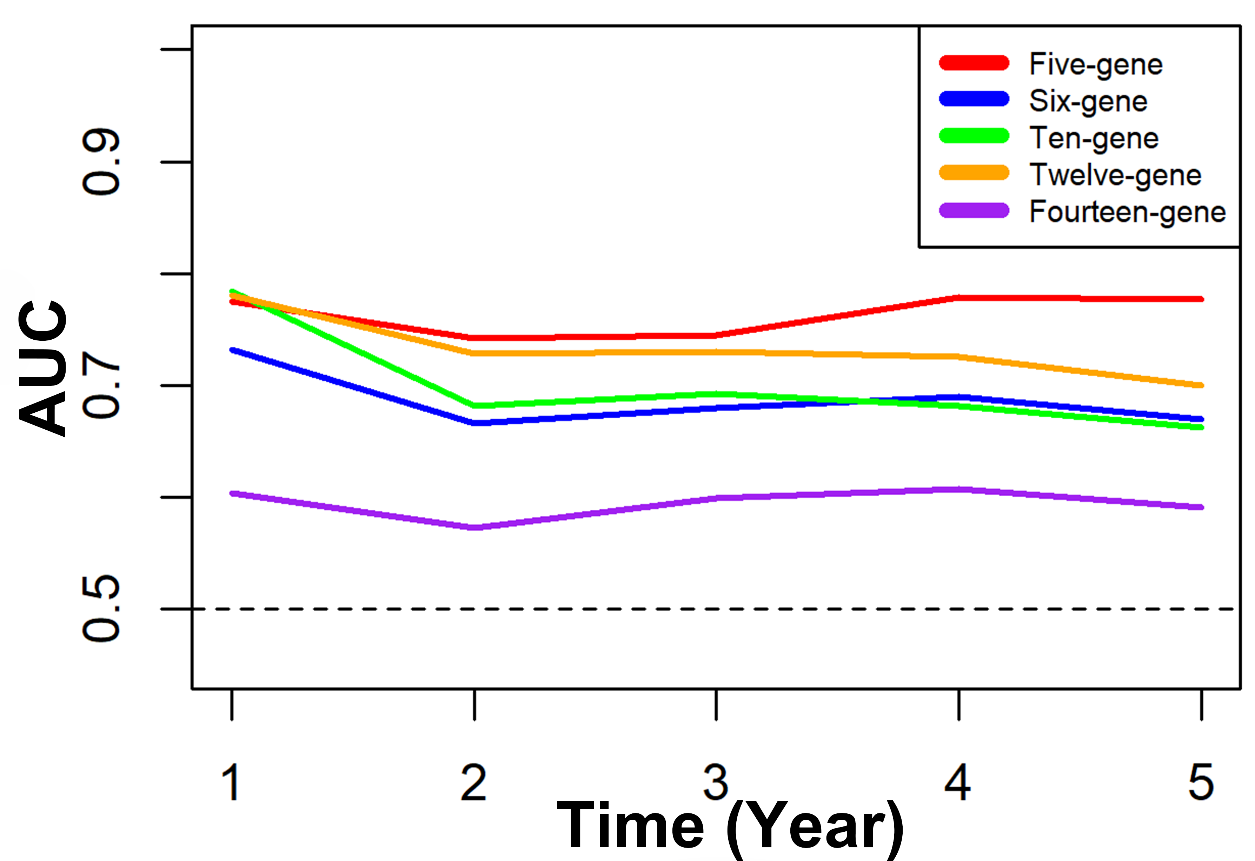


Figure S6 Comparison of the predictive power of the five-gene prognostic model with other prognostic models for survival.


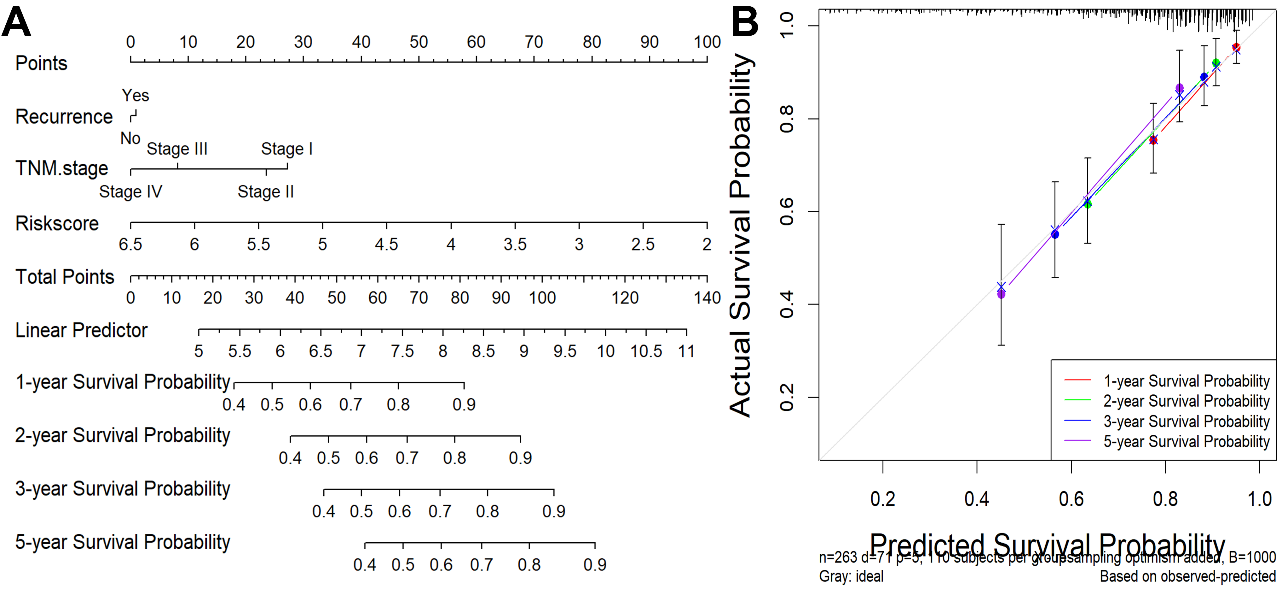


Figure S7 The predictive significance of this five-gene prognostic model was verified in nomogram.


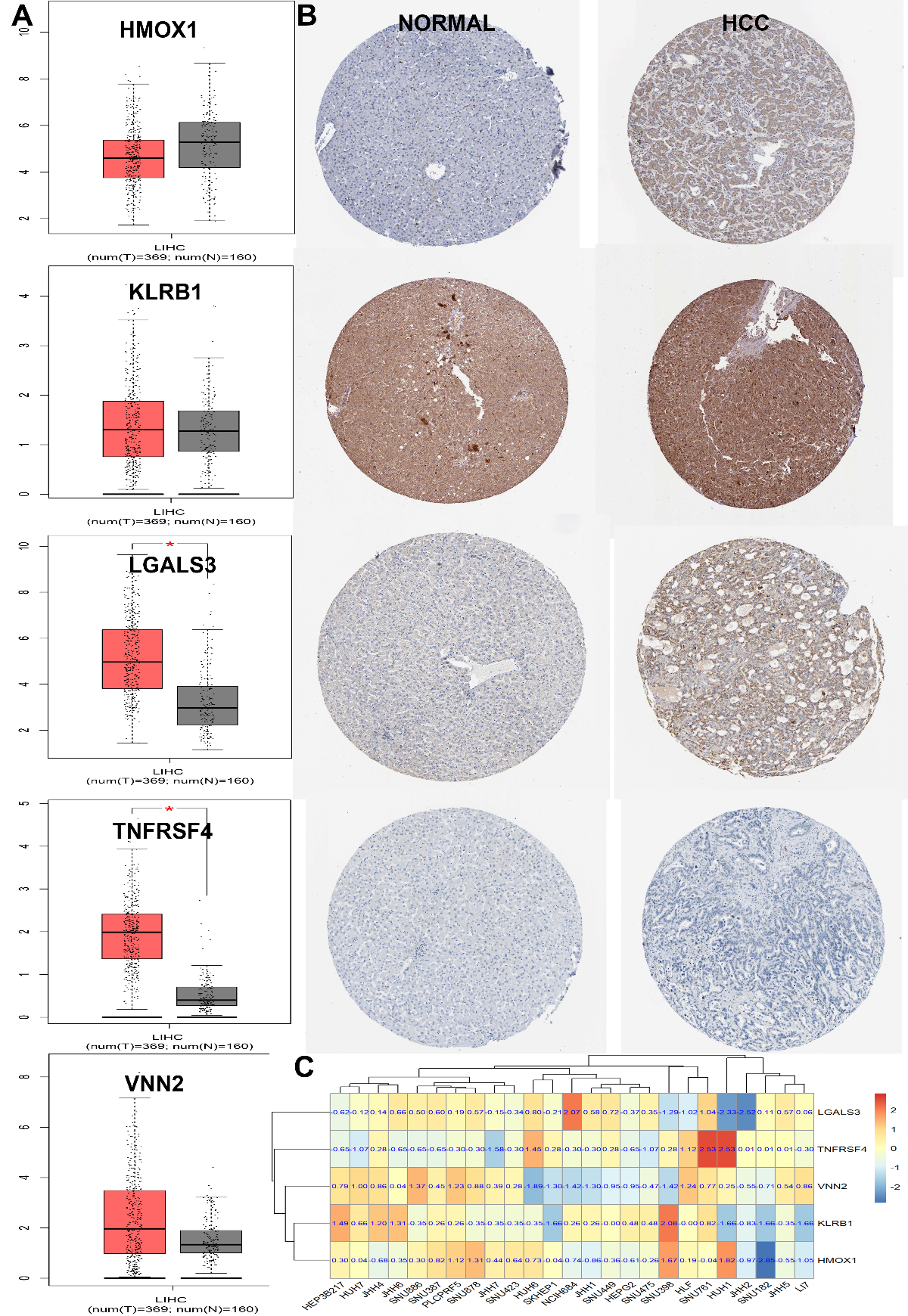


Figure S8 Expression levels of the five genes in normal and HCC tissues explored in GEPIA (A), HPA (B) and CCLE database (C).


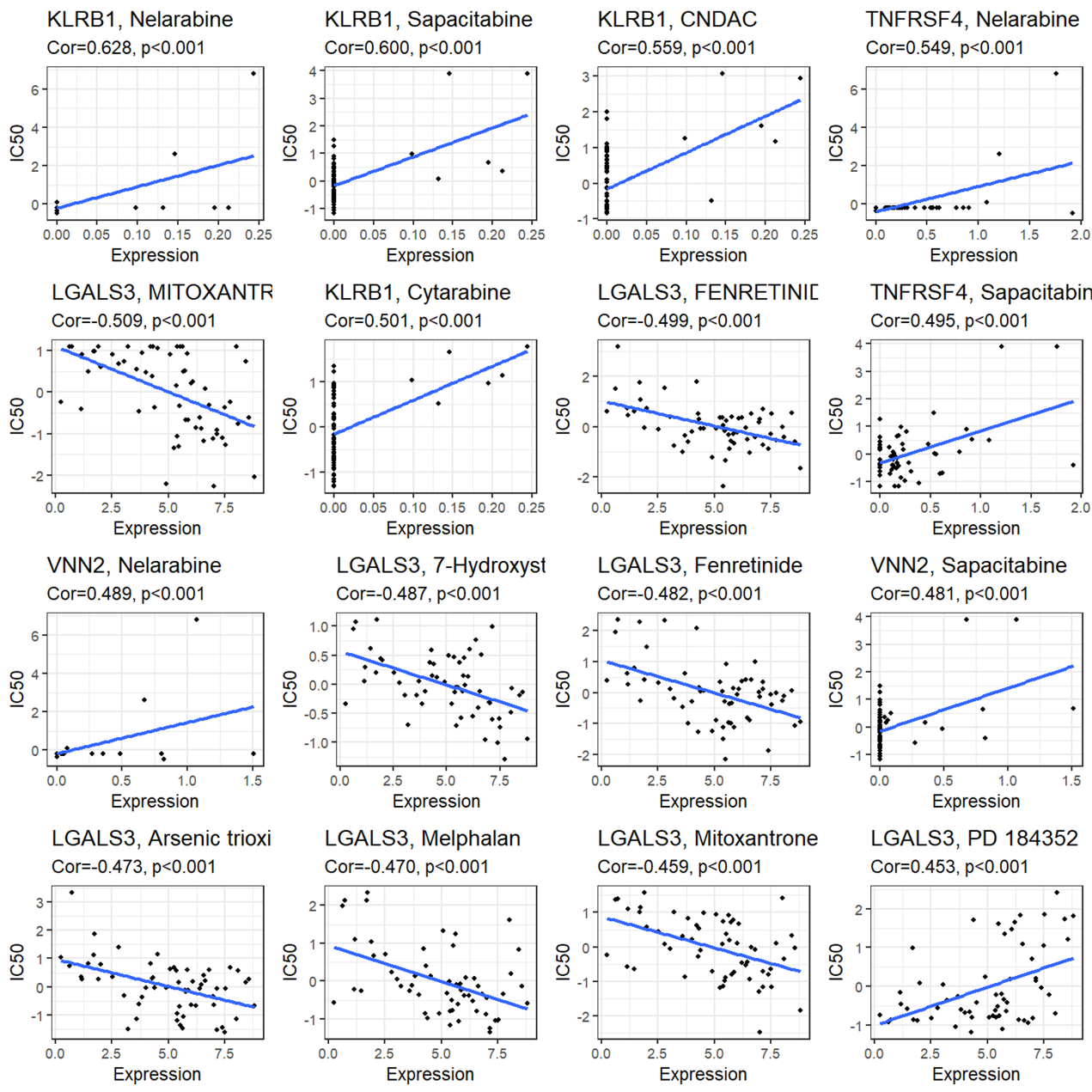


Figure S9 Top 16 most important tumor-sensitive drugs.


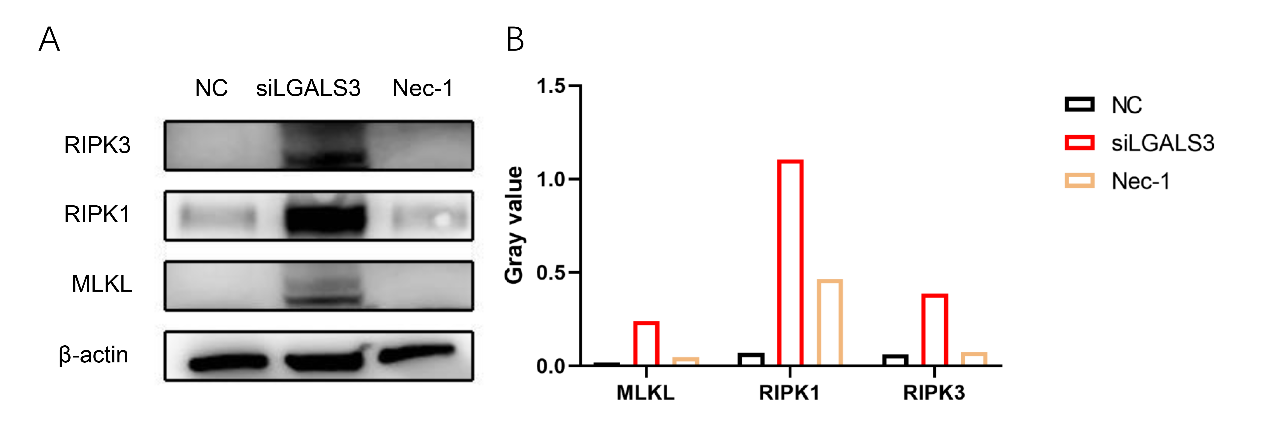


Figure S10 MLKL, RIPK1, and RIPK3 were high expression in HepG2 cell line after LGALS3 knockdown but were significantly decreased after treatment with Nec-1(A). The gray ratio of each group was calculated as follows: Gray ratio = assayed gray value of protein/ internal reference gray value of β-actin (B).

Table S1 Clinical characteristics of HCC patients involved in the study

|  | TCGA cohort  (N=365) | ICGC cohort  (N=227) | GSE14520 cohort  (N=216) |
| --- | --- | --- | --- |
| Gender Male | 119 | 61 | 187 |
| Female | 246 | 166 | 29 |
| Age ≤60 years | 173 | 49 | 178 |
| >60 years | 192 | 178 | 38 |
| Grade G1/2 | 230 |  |  |
| G3/4 | 130 |  |  |
| unknown | 5 |  |  |
| TNM Stage I/II | 254 | 140 | 167 |
| III/IV | 87 | 87 | 49 |
| unknown | 24 | 0 | 0 |
| Vascular Invasion Yes | 106 |  |  |
| No | 205 |  |  |
| unknown | 5 |  |  |
| Recurrence With tumor | 122 |  | 120 |
| Tumor free | 161 |  | 96 |
| unknown | 82 |  |  |
| Cirrhosis With | 68 |  | 198 |
| Without | 141 |  | 18 |
| unknown | 156 |  | 0 |
| HBV or HCV Infection |  |  |  |
| Yes | 149 |  | 209 |
| No | 203 |  | 6 |
| unknown | 13 |  | 1 |
| ****Child-Pugh A**** | 216 |  |  |
| B | 21 |  |  |
| C | 1 |  |  |
| unknown | 127 |  |  |

Table. S2 The sequences of the qRT-PCR primers used in this study

| Gene | Forward primer | Reverse primer |
| --- | --- | --- |
| HMOX1 | AAGACTGCGTTCCTGCTCAAC | AAAGCCCTACAGCAACTGTCG |
| VNN2 | CAGGGTGCTCGAATCATTGTG | CACGGAATCCAGTTCACCTGA |
| TNFRSF4 | GCAATAGCTCGGACGCAATCT | GAGGGTCCCTGTGAGGTTCT |
| KLRB1 | TGGCATCAATTTGCCCTGAAA | TCCAAGGGTTGACAGTGTGAG |
| LGALS3 | GTGAAGCCCAATGCAAACAGA | AGCGTGGGTTAAAGTGGAAGG |
| MLKL | AGGAGGCTAATGGGGAGATAGA | TGGCTTGCTGTTAGAAACCTG |
| RIPK1 | GGGAAGGTGTCTCTGTGTTTC | CCTCGTTGTGCTCAATGCAG |
| RIPK3 | ATGTCGTGCGTCAAGTTATGG | CGTAGCCCCACTTCCTATGTTG |
| IL10 | GACTTTAAGGGTTACCTGGGTTG | TCACATGCGCCTTGATGTCTG |
| TNF-α | GAGGCCAAGCCCTGGTATG | CGGGCCGATTGATCTCAGC |
| β-ACTIN | CGTGGGCCGCCCTAGGCACCA | TTGGCTTAGGGTTCAGGGGGG |
